# 

**Published:** 1914-06

**Authors:** 


### The Library of the

### Bristol Medico-Chirurgical Society.

*The following donations have been received since the publication of the list in March.*

*May 30th*, 1914.

L. M. Griffiths (1) .. .. .. .. .. 27 volumes.
Mr. E. W. Hey Groves (8) .. .. .. 1 volume.
Dr. R. G. Poole Lansdown (2) .. .. .. 120 volumes
Editor of *The Medical Officer* (3) .. .. .. 2 ,,
New York Surgical Society (4) .. .. .. 1 volume.
Dr. R. Shingleton Smith (5) .. .. .. 1 ,,
The Surgeon-General, United States Army (6) .. 1 ,,
J. Wright & Sons Ltd. (7) .. .. .. 2 volumes.

A framed picture has been presented by Mr. Paul Bush.

### NINETY-SECOND LIST OF BOOKS.

The titles of books mentioned in previous lists are not repeated.

The figures in brackets refer to the figures after the names of the donors, and show by whom the volumes were presented. The books to which no such figures are attached have either been bought from the Library Fund, or received through the *Journal*.

**Adler, De F. Willard and L. H.** *Anæsthesia* .. .. .. .. (2) 1891
**Beattie, C. C. Choyce and J. M.** [Eds.] *A System of Surgery* Vol. III 1914
**Binnie, J. F.** .. *Manual of Operative Surgery* (2) 2nd Ed. 1906
**Bishop, L. F.** .. *Arteriosclerosis* .. .. .. .. .. .. .. .. .. 1914
**Blackman, R. D.** [Ed.] *Dictionary of Foreign Phrases* (2) 2nd Ed. [N.D.]
**Bowlby, A. A.** .. *Surgical Pathology* .. .. .. .. .. .. (2) 1887
**Bryant, J. D.** .. *Operative Surgery.* 2 vols. (2) 3rd Ed. 1900–1
**Buxton, D. W.** .. *Anæsthetics* .. .. .. .. 5th Ed. 1914
**Cameron, Sir H. C.** *The Evolution of Wound Treatment* .. .. (2) 1907
**Carpenter, Wesley M.** *Index of the Practice of Medicine* .. .. (1) 1884
**Carrington, R. E.** .. *Dissections* .. .. .. .. .. .. .. .. (2) 1881
*Catalogue* (*Index-*) *of the Library of the Surgeon-General's Office, United States Army* .. .. 2nd Ser., Vol. XVIII (6) 1913
**Choyce and J. M. Beattie, C. C.** [Eds.] *A System of Surgery* Vol. III 1914
**Cooper, B. B.** .. *Life of Sir Astley Cooper, Bart.* 2 vols. (7) 1843
**Cunningham, G. G.** *Biographical History of England.* 16 vols. (1) 1855
**Erichsen, J. E.** .. *Surgery.* 2 vols. .. .. (2) 10th Ed. 1895
**Galton F.** .. .. *Finger Prints* .. .. .. .. .. .. .. .. (2) 1892
**Gray, H.** .. .. .. *Anatomy* .. .. .. .. .. .. (2) 10th Ed. 1883

| | | |
|---|---|---|
| **Hartenberg, P.** .. | *Neurasthenia* (Trans. by E. Playfair) .. .. | 1914 |
| **Hartshorne, H.** .. | *Essentials of Medicine* .. .. (1) 5th Ed. | 1881 |
| **Heath, C.** .. .. | *Bandaging* .. .. .. .. .. .. (2) 11th Ed. | 1897 |
| **Hilton, J.** .. .. | *Rest and Pain* .. .. .. .. .. (2) 2nd Ed. | 1877 |
| **Holden, L.** .. .. | *Landmarks* .. .. .. .. .. .. (2) 4th Ed. | 1888 |
| **Howard, R.** .. .. | *The Practice of Surgery* .. .. .. .. .. .. | 1914 |
| **Husband, H. A.** .. | *Forensic Medicine* .. .. .. (2) 5th Ed. | 1889 |
| **Husband, H. A.** .. | *The Student's Pocket Prescriber* .. 4th Ed. | 1914 |
| **Jansen, M.** .. .. | *Achondroplasia* .. .. .. .. .. .. .. .. | 1912 |
| **Kaposi, M.** .. .. | *Diseases of the Skin* .. .. .. .. .. (2) | 1895 |
| **Keith, A.** .. .. | *Human Embryology* .. .. .. .. .. (2) | 1902 |
| **Kocher, T.** .. .. | *Operative Surgery* .. .. .. .. .. .. (2) | 1894 |
| **Leaf, C. H.** .. .. | *Clinical Causes of Cancer of the Breast* .. (2) | 1904 |
| **M'Lachlan, J.** .. | *Applied Anatomy.* 2 vols. .. (2) 3rd Ed. | 1889 |
| **Macmunn, C. A.** .. | *Spectrum Analysis* .. .. .. .. .. .. .. | 1914 |
| **Mercier, C. A.** .. | *Astrology in Medicine* .. .. .. .. .. | 1914 |
| **Michels, R.** .. .. | *Sexual Ethics* .. .. .. .. .. .. .. .. | 1914 |
| **Murray, R. W.** .. | *Hare-lip and Cleft Palate* .. .. .. (2) | 1902 |
| **Napheys, G. H.** .. | *Medical Therapeutics* .. .. (1) 7th Ed. | 1880 |
| **Nash, E. H. T.** .. | *School Lighting* .. .. .. .. .. .. .. | 1914 |
| **Nettleship, E.** .. | *Diseases of the Eye* .. .. .. (2) 4th Ed. | 1887 |
| **Nisbet, W.** .. .. | *The Clinical Guide.* 4 vols. .. .. (1) | 1799–1800 |
| **Owen, E.** .. .. | *Appendicitis* .. .. .. .. .. .. .. .. | 1914 |
| **Paterson, H. J.** .. | *Hunterian Lectures on Gastric Surgery* .. (2) | 1906 |
| **Paterson, H. J.** .. | *The Surgery of the Stomach* .. .. New Ed. | 1914 |
| **Ross, E. H.** .. .. | *The Reduction of Domestic Flies* .. .. (5) | 1913 |
| **Sandow, E.** .. .. | *Strength and how to obtain it* .. .. .. (2) | [N.D.] |
| **Sharpe, J. B.** .. | *A Manual of Percussion and Auscultation* (1) | 1832 |
| **Silk, J. F. W.** .. | *Modern Anæsthetics* .. .. .. .. .. .. .. | 1914 |
| **Southam, F. A.** .. | *Regional Surgery.* Parts II, III .. (2) | 1884–86 |
| *Spiritual Healing.* | .. .. .. .. .. .. .. .. .. .. .. .. | 1914 |
| **Sturge, R. F.** .. | *Thirty Years' Weather at Bristol,* 1860–89 (1) | 1890 |
| **Sturge, R. F.** .. | *The Weather at Clifton from* 1890 *to* 1900 (1) | 1901 |
| **Thomson, J. A.** .. | *Outlines of Zoology* .. .. .. .. .. 6th Ed. | 1914 |
| **Treves, F.** .. .. | *System of Surgery.* 2 vols. .. .. .. (2) | 1895 |
| **Wilks, S.** .. .. | *Lectures on the Specific Fevers* .. .. (2) | 1873–74 |
| **Willard and L. H. Adler, De F.** | *Anæsthesia* .. .. .. .. .. (2) | 1891 |
| **Wilson, G.** .. .. | *Handbook of Hygiene* .. .. (2) 6th Ed. | 1886 |
| **Yeo, G. F.** .. .. | *Physiology* .. .. .. .. .. .. .. .. (2) | 1884 |
| **Zorraquin, G.** .. | *Le Traitement des Sténoses aigues du Larynx* | 1914 |

### TRANSACTIONS, REPORTS, JOURNALS, &c.

| | | |
|---|---|---|
| American Association of Genito-Urinary Surgeons, Transactions of the .. .. .. .. .. .. .. .. .. .. .. .. .. .. .. .. .. | Vol. VIII | 1913 |
| American Journal of Ophthalmology .. .. .. .. .. | Vol. XXX | 1913 |
| American Journal of the Medical Sciences .. .. .. | Vol. CXLVI | 1913 |
| American Journal of Surgery .. .. .. .. .. .. | Vol. XXVII | 1913 |

| | | |
|---|---|---|
| American Medicine .. .. .. .. .. .. .. .. | N.S., Vol. VIII | 1913 |
| American Pediatric Society, Transactions of the .. | Vol. XXV | 1913 |
| Annales de Dermatologie .. .. .. .. .. .. .. | Bd. IV | 1913 |
| Annals of Tropical Medicine .. .. .. .. .. .. .. .. | Vol. VI | 1912 |
| Archiv für Klinische Chirurgie .. .. .. .. .. .. .. | Bd. CII | 1913 |
| British Journal of Dermatology, The .. .. .. .. | Vol. XXV | 1913 |
| British Journal of Surgery, The .. .. .. .. .. | Vol. I | 1913–14 |
| Bulletin de l'Académie de Médecine .. .. | Tom. LXIX, LXX | 1913 |
| Canadian Medical Association Journal, The .. .. | Vol. III | 1913 |
| Clinical Journal, The .. .. .. .. .. .. .. .. | Vol. XLII | 1913 |
| Deutsche Zeitschrift für Chirurgie .. .. .. .. .. | Bd. CXXV | 1913 |
| Edinburgh Medical Journal .. .. .. .. .. .. | N.S., Vol. XI | 1913 |
| English Catalogue of Books, The .. .. .. .. .. .. .. .. | | 1913 |
| Gazette des Hopitaux .. .. .. .. .. .. .. .. .. .. | | 1913 |
| Gazette hebdomadaire des Sciences médicales de Bordeaux .. .. | | 1913 |
| Hospital—St. Bartholomew's Hospital Reports .. | Vol. XLIX | 1914 |
| Hospitals and Charities, Burdett's .. .. .. .. .. .. .. | | 1914 |
| Indian Medical Gazette, The .. .. .. .. .. .. .. | Vol. XLVII | 1912 |
| International Abstract of Surgery .. .. .. .. .. | Vol. XVII | 1913 |
| Jahreskurse für Ärtzliche Fortbildung .. .. .. .. .. .. | (8) | 1913 |
| Journal of Experimental Medicine, The .. .. .. | Vol. XVIII | 1913 |
| Journal of Medical Research, The.. .. .. .. | Vol. XXVIII | 1913 |
| Journal of Mental Science .. .. .. .. .. .. .. | Vol. LXIX | 1913 |
| Journal of Nervous and Mental Diseases, The .. .. | Vol. XL | 1913 |
| Journal of the American Medical Association .. .. | Vol. LXI | 1913 |
| Journal of the Royal Sanitary Institute .. .. | Vol. XXXIV | 1913 |
| Journal of Tropical Medicine, The .. .. .. .. | Vol. XVI | 1913 |
| Lancet, The .. .. .. .. .. .. .. .. .. .. .. | Vol. II for | 1913 |
| Medical Annual, The .. .. .. .. .. .. .. .. .. .. .. .. | | 1914 |
| Medical Chronicle, The .. .. .. .. .. .. .. .. | Vol. XXV | 1913 |
| Medical Directory, Nisbet's .. .. .. .. .. .. .. .. .. .. | (2) | 1912 |
| Medical Officer, The .. .. .. .. .. .. .. | (3) Vols. IX, X | 1913 |
| Medical Record, The .. .. .. .. .. .. .. | Vol. LXXXIV | 1913 |
| Medical Review, The .. .. .. .. .. .. .. .. | Vol. XVI | 1913 |
| Medical Society's Transactions, The .. | Vols. XXXIV–XXXVI | 1911–13 |
| Medical Society's Transactions, General Index to | Vols. I–XXXI | 1911 |
| New York Surgical Society, Transactions of the .. | (4) Vol. I | 1912 |
| Press Guide, Willing's .. .. .. .. .. .. .. .. .. .. .. | | 1914 |
| Progressive Medicine .. .. .. .. .. .. .. .. .. | Vol. I | 1914 |
| Revue de Chirurgie .. .. .. .. .. .. .. .. | Tom. XLVIII | 1913 |
| Revue de Thérapeutique .. .. .. .. .. .. .. .. .. .. | | 1913 |
| Royal Society of Medicine, Proceedings of the .. .. | Vol. VI | 1913 |
| Surgery, Gynecology and Obstetrics .. .. .. .. | Vol. XVII | 1913 |
| Therapeutic Gazette .. .. .. .. .. .. .. | Vol. XXXVII | 1913 |
| West London Medical Journal .. .. .. .. .. | Vol. XVIII | 1913 |
| Wiener klinische Wochenschrift .. .. .. .. .. .. .. .. | | 1913 |
| Zeitschrift für Urologie .. .. .. .. .. .. .. .. | Bd. VII | 1913 |

### Local Medical Notes.

**University of Bristol.**—Students of the University have recently passed the under-mentioned examinations :—

UNIVERSITY OF LONDON.—*Second Examination for Medical Degrees, Part I*—R. B. Britton, D. W. Evans, G. E. Tilsley. *Part II :* C. C. Chesterman.

F.R.C.S.—*Primary Examination :* A. W. Adams, F. K. Hayman.

CONJOINT EXAMINING BOARD OF ENGLAND.—*Final Examination, Medicine :* R. I. Dacre, H. B. Logan. *Surgery :* W. E. Taylor.* *Midwifery :* G. M. Campbell.*

LICENCE IN DENTAL SURGERY, R.C.S. ENGLAND.—*Mechanical Dentistry and Dental Metallurgy :* G. M. Haydon, W. S. Hazell, R. V. Knight, W. H. B. Stride, R. H. Waters, A. E. Young. *Dental Metallurgy :* S. W. A. Davis. *Mechanical Dentistry :* L. B. James. *Final Examination, Part I :* W. A. Culverwell, E. E. Wookey, E. W. Joyner, W. J. Milton.

The Smith-Turner Scholarship in Mechanical Dentistry has been awarded to R. Waters.

ROYAL NAVAL MEDICAL SERVICE.—At the recent examination for appointments as Acting Surgeons, Dr. H. B. Parker, a former Bristol student, was placed second.

### APPOINTMENTS.

G. M. Campbell, M.R.C.S., L.R.C.P., has been appointed House Surgeon to the Bristol Royal Infirmary.

C. C. Harrison, M.B., B.S. Lond., has been appointed House Surgeon to the Bristol Royal Infirmary.

E. S. Sowerby, M.B., B.S. Lond., has been appointed House Physician at the Bristol Royal Infirmary.

W. E. Taylor, M.R.C.S., L.R.C.P., has been appointed Casualty House Surgeon at the Bristol General Hospital.

R. B. Johnson, M.R.C.S., L.R.C.P., has been appointed First House Physician at the Bristol General Hospital.

B. J. L. Fayle, M.R.C.S., L.R.C.P., has been appointed House Surgeon at the Bristol General Hospital.

R. Creasey, M.R.C.S., L.R.C.P., has been appointed Second House Physician at the Bristol General Hospital.

* Denotes qualification.

